# The Phenolic Content, Antioxidative Properties and Extractable Substances in Silver Fir (*Abies alba* Mill.) Branches Decrease with Distance from the Trunk

**DOI:** 10.3390/plants11030333

**Published:** 2022-01-26

**Authors:** Katja Schoss, Rebeka Benedetič, Samo Kreft

**Affiliations:** Department of Pharmaceutical Biology, Faculty of Pharmacy, University of Ljubljana, Aškerčeva 7, 1000 Ljubljana, Slovenia; rebeka.benedeti@gmail.com (R.B.); samo.kreft@ffa.uni-lj.si (S.K.)

**Keywords:** silver fir branch extract, lignans, ABTS, DPPH

## Abstract

Silver fir (*Abies alba* Mill.) is one of the most common and valuable conifer tree species in Central Europe, with well-established usage in the construction and furniture industries, as well as the food, health products, pharmaceuticals and cosmetics industries. Silver fir branch extract, a mixture of antioxidative phenols, is produced industrially as a food supplement with a wide range of therapeutic properties. This study investigates optimization of the production of silver fir branch extract by researching its antioxidant activity (ABTS and DPPH assay), phenol content (Folin-Ciocalteu assay), lignan content (HPLC) and extractable content at various distances from the trunk (0–80 cm). The antioxidative activity, phenol content and extractable content decreased from the proximal to the distal part of the branch. The decrease in ABTS assay activity was 51%, and that of the DPPH assay was 52%; the decrease in total phenol content was 35–40%; and the decrease in lignan content was 91%. The extractable matter content was reduced by 40%. Data gained in the study herein justifies the importance of researching existing and industrially produced plant extracts for further optimization of the final product. Results shows that industry can also produce extracts with elevated content of lignans with the use of short proximal parts of the branches.

## 1. Introduction

Silver fir (*Abies alba* Mill.) is one of the most common and valuable conifer tree species in Central Europe for historical and economic reasons [1]. Its usage for consumption started over a century ago in the production of, for example, silver fir beer [2]. It is a medicinal plant that is used in the food, health products, pharmaceuticals and cosmetics industries [3,4,5]. Research and publications provide scientific evidence to support claims that silver fir phytochemicals provide health benefits to consumers. One of the largest groups of phytochemicals comprises phenolic chemicals, which are universally present in higher plants [6]. In addition, bioactive phenols such as phenolic acids and flavonoids have attracted special attention because they can protect the human body from oxidative stress, which may cause numerous diseases including cancer, cardiovascular problems and aging [7,8,9,10]. Furthermore, polyphenols could also improve insulin resistance [11] and be used as radioprotective agents [12].

The diverse benefits of phenolic antioxidants have led to considerable consumer interest in phenol-containing extracts. Examples of such free radical scavengers are extracts from the bark and wood of different conifer species, the most studied of which is commercially known as Pycnogenol^®^ (extract from *Pinus maritima* Mill.) [13,14,15,16], a mixture of phenolic compounds. Similar extracts are produced from silver fir, such as that from its branches (trade name Belinal^®^). This extract is a complex combination of antioxidative phenols that can be divided into three main types: lignans, flavonoids and phenolic acids, which have antioxidative [17,18], vasoprotective [19], cardioprotective [20] and antidiabetic activities [21].

Although silver fir extract is already on the market, it is beneficial to research the extract further in order to help the industry to additionally optimize it. This is especially true since the composition of extracts can vary drastically depending on the plant location [22], time of year [23,24], the stage of the plant (young plant, plant at the time of flowering etc.) [25,26,27] and the plant part that is extracted [28,29]. Previous studies have also shown that the phenol content in the trunk drastically varies with respect to height [30], and between sapwood, heartwood, knotwood and branchwood [31]. In this work, we analyzed the lignan content, total phenols and antioxidant activity in the branch in relation to the distance from the trunk.

## 2. Results and Discussion

### 2.1. Optimization of the Extraction Conditions

The optimal extraction parameters were found to be an extraction temperature of 100 °C, extraction time of 90 min and water:sample ratio of 1:10, which were the maximum temperature, time and water:sample ratio used, meaning that the phenols found in silver fir branches are relatively stable components. Larger parameters were not tested, as they would not be efficient in industrial conditions. The differences in antioxidant activity and phenolic content in extracts obtained under different conditions were not large, meaning that the extraction efficiency was high and robust.

### 2.2. Phenolic Compound Content in Relation to the Distance from the Trunk

**Figure 1:**
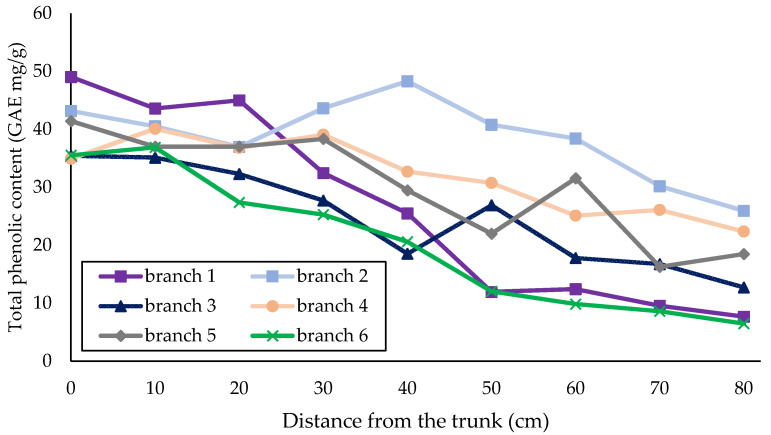
Total phenolic content of six branches determined with FC reagent.

The total phenolic compounds of SFBE were measured spectrophotometrically with FC reagent. The total phenolic content is expressed in gallic acid equivalents. As the method is not very selective, it gives an excellent relative comparison between samples but does not necessarily reflect the actual absolute amount of phenolic content in the extract. Figure 1 presents the total phenol content in consecutive segments of individual branches where a steady decrease was noticed. The average decline in phenol content was 61% (the phenol content was 61% lower in the distal part (at a distance of 80 cm from the trunk) than in the proximal part (the sample at 0 cm)). The largest decrease in phenol content was determined on branch 1 (84.4%) In branches 2 and 4, the content at distal part remains 2× higher than in the branch with lowest content (branch 6), so the phenolic activity decreases by approximately 35–40% with increasing distance from the trunk. The mean phenol content of all samples (28.7 mg/g) was also comparable with that of the industrial-milled branch sample (19.4 mg/g); thus, the industrial sample contained slightly fewer phenols, which can be attributed to random sampling of the branches.

### 2.3. Antioxidant Activity of Extracts

**Figure 2:**
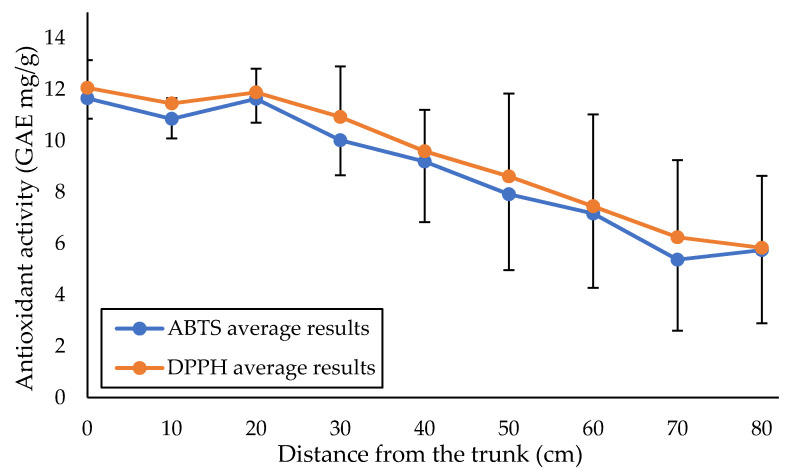
Spectrophotometric determination of antioxidants (ABTS and DPPH). Both measurements decrease significantly with the distance (Pearson correlation R < −0.95, *p* < 0.001).

The antioxidant activity of SFBE was evaluated using a spectrophotometric test with ABTS and DPPH, which are both indirect methods for antioxidant activity evaluation. Both methods are single-electron transfer methods, where colored persistent radicals are reduced via antioxidant, and the color in the solution changes [32,33]. Even if the mechanism of both assays are similar, some substances can give strong signal with one assay and weak signal with other assay, and the opposite can be the case in other substances, as shown in the previous study by Benković et al. [18]. The results of both tests in this study were found to be comparable and are shown in Figure 2. The antioxidant activity steadily declined toward the distal part of the branch, with a 52% drop in the DPPH (from 12.1 ± 1.1 mg/g GAE at 0 cm to 5.8 ± 2.8 mg/g GAE at 80 cm) and a 51% decrease in the ABTS (from 11.7 ± 0.8 mg/g GAE at 0 cm to 5.7 ± 2.9 mg/g GAE at 80 cm). The measurements for the milled branches obtained from the factory were comparable (6.40 and 6.51 mg/g GAE for the ABTS and DPPH tests). The difference between the average of all SFBE samples and industrial samples was only 28% for the ABTS test and 30% for the DPPH test.

### 2.4. Lignan Content

Six lignans previously identified in the SFBE were quantified with HPLC [18]. The content of all determined lignans decreased from the proximal part toward the distal part of the branch (Table 1). The highest difference was in secoisolariciresinol, with only 5% of the content at 80 cm compared with that next to the trunk, followed by isolariciresinol, lariciresinol, pinoresinol, matairesinol and hydroxymatairesinol with 8%, 11%, 12%, 16% and 44% remaining relative content, respectively. This reduction in lignan content (91% decrease in total) is much higher than the reduction in antioxidant activity (51–52% decrease), which implies that there are antioxidants other than lignans present in branches whose content does not decrease with the distance from the trunk as much as lignans do. The ratio of lignans in studied samples did not change along the branch and was comparable to that in the literature [18], with secoisolariciresinol at the highest concentration (on average) of 56% ± 10.3, followed by lariciresinol (13% ± 1.8), isolariciresinol (12% ± 2.7), hydroxymatairesinol (9% ± 6.6), pinoresinol (4% ± 0.9) and matairesinol (4% ± 1.2). This indicates that the ratio is generally typical for the silver fir.

### 2.5. Determination of the Extractable Content

Similar to other parameters, the extractable matter content also showed a reduction from the proximal to the distal part of the branch. As the distance from the trunk increased, there was less extractable matter in the branch, and the antioxidant and phenolic activities also decreased. As shown in Table 2, the extractable content decreased by approx. 40% along the branch, which is comparable but not equal to the decreases in total phenolic content (61%) and antioxidant activity (ABTS decrease of 51%, DPPH decrease of 52%) along the branch. We assume that in addition to phenols and lignans, as their largest share, some other extractable components also decrease with distance from the trunk. One possible explanation is that these compounds might be polysaccharides, which are present to a greater extent in aqueous extracts of wood [34].

### 2.6. Sapwood and Heartwood Area and Proportions

The area and proportion of hardwood are shown in Table 3. It was observed that the surface area of heartwood in branches 1, 2 and 5 did not change largely along the branch, while the area of branches 3, 4 and 6 decreased by more than 30 cm^2^ (Pearson correlation R < −0.90). The share of hardwood in the branches increased in the first few reels and then decreased; with the exception of branch 3, the proportion of heartwood at 80 cm was never lower than that at 0 cm. A correlation between the heartwood share in samples and the antioxidant activity, phenol content, secoisolariciresinol and extractable content was calculated, as a positive correlation was observed in the literature [35]. In this study, the correlation was negative and statistically significant for the DPPH test (R = −0.318, *p* = 0.02) and secoisolariciresinol content (R = −0.383, *p* = 0.006) and not significant for the FC test (R = −0.207, *p* = 0.15), ABTS test (R = −0.225, *p* = 0.1) or extractable content (R = −0.180, *p* = 0.20). Results in this study differ from previous observations, and the conclusion that heartwood is richer in phenols and more pharmacologically active than sapwood cannot be made with this data alone.

## 3. Materials and Methods

### 3.1. Chemicals

ABTS (2,2′-azinobis-(3-ethylbenzthiazoline-6-sulfonic acid)) (CAS: 30931-67-0) and DPPH (2,2-diphenyl-1-picrylhydrazyl) (CAS: 1898-66-4) were purchased from Sigma-Aldrich^®^ (Darmstadt, Germany); gallic acid, potassium peroxodisulfate and Na_2_CO_3_ were purchased from Fluka (Darmstadt, Germany); methanol was purchased from Carlo Erba (Milan, Italy); and Folin–Ciocalteu (FC) reagent was purchased from Merck (Darmstadt, Germany). The solvents used for the high-performance liquid chromatography (HPLC) were of HPLC-grade purity; methanol, water and acetonitrile were obtained from J. T. Baker (Gliwice, Poland) and trifluoroacetic acid was obtained from Fisher Scientific (Loughborough, U.K.). Reference standard compounds were used for confirmation of identity and HPLC quantification: matairesinol (CAS: 580-72-3), (+)-isolariciresinol (CAS: 549-29-8) and (−)-hydroxymatairesinol (CAS: 347359-71-1) were obtained from Sigma-Aldrich^®^ (Darmstadt, Germany); secoisolariciresinol (CAS: 145265-02-7) was obtained from Fluka (Darmstadt, Germany); and (+)-lariciresinol (CAS: 27003-73-2) and (+)-pinoresinol (CAS: 487-36-5) were obtained from PhytoLab (Dutendorfer, Germany).

### 3.2. Plant Material

**Figure 3:**
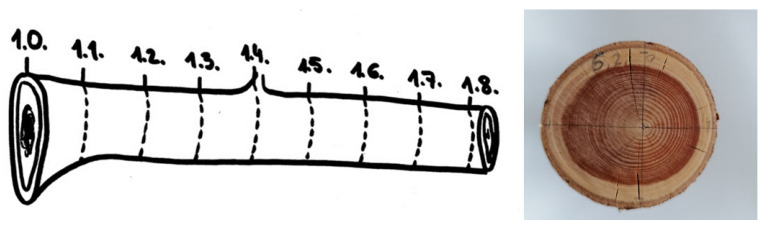
**Left**—a presentation of where silver fir samples of sawdust were collected from the branch; **right**—a presentation of how the sapwood and hardwood measurements were taken.

Six branches (cca 85 cm long and 4–9.3 cm wide) of silver fir (*Abies alba* Mill.) were obtained from Kočevje, Slovenia, in December 2020. Silver fir is the only species of fir growing in Slovenia and was identified by its morphological features, such as needles with notched tips and two greenish-white bands below, and by its typically white wood and commonly grown habitat. Branch samples are deposited in the herbarium of the Department of Pharmaceutical Biology, Faculty of Pharmacy, University of Ljubljana under the voucher number 2021-12. Sawdust was prepared from the branches using a circular saw. For further research, sawdust from nine different parts of the branch was obtained. The first sample was taken immediately next to the trunk (proximal part of the branch), and each subsequent part was 10 cm away from the previous one (Figure 3—left). The last sample of the branch was taken 80 cm from the trunk (distal part). Milled branch mix used for the industrial preparation of silver fir branch extract (SFBE, brand name Belinal^®^) was tested for comparison. The obtained sawdust was collected in paper bags, stored in a dry and dark placed at ambient temperature and used within a week.

### 3.3. Measurement of Sapwood and Hardwood

At each end of the 10 cm reel where the samples were taken, the hardwood (dark-colored wood) and the total branch diameters were measured along the widest and narrowest axes (Figure 3—right). The area of hardwood and total branch cross section and their ratio were determined by calculating the average of the diameter of the widest and narrowest axes and using the formula for the area of a circle (πr^2^).

### 3.4. Optimization of Extraction Procedures

The parameters for the optimization of extraction were selected based on the internal industrial extraction guidelines for the preparation of SFBE: water solvent (ratio of water: branches of 1:5) was heated to 70–90 °C for 30–60 min. The obtained samples were extracted using water solvent at different temperatures (60, 80, 100 °C) and extraction times (30, 60, 90 min) in an ultrasonic bath (Bandelin Sonorex RK 52 H, Bandelin electronic GmbH & Co. KG, Berlin, Germany). The volume of solvent was 5, 7.5 or 10 mL per 1 g of sample. Twenty extracts were prepared with different combinations of these three variables obtained with Design-Expert^®^ 7.0 software (Stat-Ease, Inc., Minneapolis, U.S.A.) (central composite design (CCD) with the responses of the dependent variables). Liquid extracts were separated by filtration and kept at 4 °C until further analysis of the antioxidant and phenol content over a maximum period of two days. An optimization experiment was carried out using response surface methodology for the extraction of total phenols and the ABTS and DPPH test results.

### 3.5. Antioxidant Activity

The ability of extracts to neutralize free radicals was assessed using the DPPH and ABTS radical methods. The DPPH method was performed according to Sharma [36], with slight modifications. A total of 3 mL of DPPH radical solution (80 mg/L in methanol) was added to 3 mL of 300× diluted extract in water. In parallel, a control sample containing the same volume of water instead of the extract was prepared. The obtained mixtures were allowed to stand for 30 min in the dark at ambient temperature. The absorbance of the samples was read at λ = 517. Each sample was measured three times. The ABTS method used was described by Re [37]; 4875 µL of diluted ABTS was added to 125 µL of 40× diluted extract in water and allowed to stand for 6 min in the dark at ambient temperature. Each sample was measured three times. All spectrophotometric measurements were performed on a UV/Vis spectrophotometer (Nanocolor, Macherey-Nagel, Düren, Germany). The DPPH and ABTS activity results were expressed in mg of gallic acid equivalents per g of air-dried branch wood material (mg GAE/g).

### 3.6. Determination of Phenolic Compounds

#### 3.6.1. Spectrophotometric Determination of Phenolic Compound Content

The amount of total phenols was determined using the Folin–Ciocalteu method described by Fernandes [38] with slight modification. Briefly, an aliquot of 40 µL of sample solution was diluted in 11,960 µL of water (to obtain absorbance in the range of the prepared calibration curve), mixed with 600 µL of FC reagent and allowed to stand for 3 min in the dark. Then, 1200 µL of 20% Na_2_CO_3_ in water was added and incubated for 60 min in the dark before the absorbance of the reaction mixture was read at 750 nm. Gallic acid was used as a standard, and the results were expressed in mg of gallic acid equivalents per g of air-dried branch wood material (mg GAE/g).

#### 3.6.2. HPLC Analysis

To identify and quantify individual lignans, an HPLC system (Shimadzu Prominence, Kyoto, Japan) was used as described by Benković [17]. The amount of each lignan in the original sample was calculated using calibration curves of standard compounds (where y is the peak height and x is the concentration of the lignan (mg/mL)):

Isolariciresinol: y = 966x − 2565 R^2^ = 0.989
Hydroxymatairesinol: y = 528x − 2168 R^2^ = 0.985
Secoisolariciresinol: y = 2911x + 58134 R^2^ = 0.995
Lariciresinol: y = 752x − 2020 R^2^ = 0.981
Pinoresinol: y = 599x − 1317 R^2^ = 0.985
Matairesinol: y = 519x + 18 R^2^ = 0.999

### 3.7. Determination of the Content of Extractable Matter

The number of extractable substances was determined by lyophilization (Lio 2000, Kambič, Semič, Slovenia) of the water extracts with programmed primary and secondary drying temperatures of −20 °C and 20 °C (vacuum: 0.5012 mbar), respectively.

### 3.8. Statistical Analysis

Statistical analyses were performed using Excel software Office Home and Design-Expert^®^ 7.0 software (Stat-Ease, Inc., Minneapolis, U.S.A.). The average values of measurements within the groups are presented as means, and their variability by standard deviation. Correlation is presented by Pearson coefficient. Significance level *p* < 0.05 was used.

## 4. Conclusions

This paper emphasizes the importance of researching existing plant extracts to help the industry produce optimized products. In the case of silver fir branch extract, a reduction in the content of total extractable substances (40% decrease), phenols (61% decrease), lignans (91% decrease) and antioxidative activity (51–52% decrease) along the branch was found. Since phenol content and antioxidative activity in branches decline approximately in parallel with total extractable substances, the quality (regarding phenol content and antioxidative activity) of the produced extract does not greatly depend on the branch part used.

On the other hand, the short proximal parts of the branches would need to be used in order to produce extracts with elevated content of lignans.

Whether it is reasonable to use longer or shorter branches in industrial-scale extraction depends on parameters that were not investigated in this study (such as the cost of water evaporation, cost of branch collection, etc.) and this would be an interesting topic for further research.

## Figures and Tables

**Table 1:** Content of individual quantified lignans (mg/g) compared with that at the proximal part of the branch.

Distance from the Trunk (cm)	Isolariciresinol	Hydroxymatairesinol	Secoisolariciresinol	Lariciresinol	Pinoresinol	Matairesinol
0	1.76 ± 0.35	0.54 ± 0.11	5.95 ± 1.41	1.34 ± 0.19	0.49 ± 0.12	0.32 ± 0.08
10	1.20 ± 0.33	0.44 ± 0.08	5.30 ± 1.38	0.97 ± 0.37	0.37 ± 0.15	0.25 ± 0.05
20	0.73 ± 0.28	0.37 ± 0.08	3.89 ± 1.84	0.67 ± 0.26	0.26 ± 0.13	0.17 ± 0.10
30	0.89 ± 0.30	0.49 ± 0.13	5.25 ± 2.31	1.10 ± 0.32	0.32 ± 0.16	0.33 ± 0.07
40	0.49 ± 0.31	0.36 ± 0.18	3.17 ± 2.21	0.69 ± 0.18	0.18 ± 0.10	0.23 ± 0.08
50	0.39 ± 0.49	0.29 ± 0.21	2.23 ± 2.74	0.46 ± 0.14	0.14 ± 0.12	0.11 ± 0.11
60	0.36 ± 0.32	0.34 ± 0.26	2.57 ± 2.28	0.54 ± 0.15	0.15 ± 0.10	0.22 ± 0.16
70	0.15 ± 0.10	0.25 ± 0.21	0.72 ± 1.24	0.26 ± 0.07	0.07 ± 0.04	0.10 ± 0.07
80	0.14 ± 0.15	0.24 ± 0.30	0.29 ± 1.03	0.15 ± 0.06	0.06 ± 0.04	0.05 ± 0.06
Industrial sample	0.26	0.21	0.50	0.17	0.09	0.04

**Table 2:** Extractable matter content (mean values and standard deviations) in the branches at different distances from the trunk.

Distance from the Trunk (cm)	Average Extractable Content (mg/g)	%
0	76.5 ± 11.6	100
10	67.5 ± 9.8	88
20	71.9 ± 14.7	94
30	62.8 ± 11.1	82
40	60.3 ± 15.4	79
50	52.4 ± 15.2	69
60	49.5 ± 12.5	65
70	43.8 ± 13.4	57
80	45.3 ± 16.2	59

**Table 3:** Sapwood and heartwood areas and proportions of silver fir branches.

Distance from the Trunk (cm)	Branch 1		Branch 2		Branch 3		Branch 4		Branch 5		Branch 6	
/	Heartwood Area (cm^2^)	Heartwood Proportion (%)	Heartwood Area (cm^2^)	Heartwood Proportion (%)	Heartwood Area (cm^2^)	Heartwood Proportion (%)	Heartwood Area (cm^2^)	Heartwood Proportion (%)	Heartwood Area (cm^2^)	Heartwood Proportion (%)	Heartwood Area (cm^2^)	Heartwood Proportion (%)
0	29.2	28.6	50.3	22.4	44.2	21.4	73.9	32.9	62.2	43.5	95.0	59.2
10	28.3	40.7	46.6	42.6	41.9	38.3	67.9	60.1	59.4	64.9	78.5	78.3
20	25.5	37.6	47.8	46.8	36.3	36.2	60.8	62.9	47.8	51.2	80.1	79.9
30	23.8	40.0	45.4	53.4	32.2	33.9	47.8	50.3	44.2	50.1	78.5	74.3
40	30.2	52.0	52.8	57.6	28.3	31.4	44.2	44.8	55.4	65.2	75.4	75.2
50	33.2	58.5	41.9	59.0	30.2	29.6	40.7	43.6	54.1	64.9	72.4	74.8
60	35.3	54.2	37.4	51.7	21.2	24.5	29.2	32.5	58.1	55.0	69.4	71.7
70	29.2	55.3	44.2	43.3	25.5	30.6	27.3	30.4	52.8	61.0	66.5	71.2
80	29.2	51.5	51.5	44.8	11.3	13.9	29.2	33.8	58.1	65.8	65.0	69.7
Pearson correlation (R)	0.41	0.85	−0.25	0.45	−0.95	−0.48	−0.97	−0.57	−0.05	0.55	−0.92	0.04

## Data Availability

Not applicable.
